# Role of Endogenous Myoglobin in Anthracycline Response in Breast Cancer

**DOI:** 10.3390/biom16071055

**Published:** 2026-07-18

**Authors:** Ilona Rybinska, Andreas Petry, Thomas Hankeln, Thomas A. Gorr, Gaetano Cairo

**Affiliations:** 1 Department of Medical Biotechnology and Translational Medicine, University of Milan, 20133 Milan, Italy; 2 Experimental and Molecular Pediatric Cardiology, German Heart Center, TUM University Hospital, Technical University of Munich, 80636 Munich, Germany; petry@dhm.mhn.de; 3 Institute of Organismic and Molecular Evolution, Molecular Genetics and Genome Analysis, Johannes Gutenberg University, 55128 Mainz, Germany; hankeln@uni-mainz.de; 4 Department of Veterinary Physiology, University of Zurich, CH-8057 Zurich, Switzerland; tgorr@access.uzh.ch; 5 Department of Biomedical Sciences for Health, University of Milan, 20133 Milan, Italy

**Keywords:** anthracyclines, breast cancer, myoglobin, hypoxia, chemotherapy resistance

## Abstract

Anthracyclines such as doxorubicin (DOX) remain central components of breast cancer (BC) chemotherapy, although their efficacy is frequently limited by drug resistance. Myoglobin (MB), an oxygen-binding heme protein expressed in breast tumors, has been implicated in the detoxification of DOX in cardiomyocytes, but its role in BC remains unclear. Using MB-expressing and MB-knockout (MBKO) MDA-MB-468 BC cells, we demonstrate that MB confers hypoxia-dependent resistance to DOX. Under hypoxia, MB-expressing cells exhibited reduced intracellular DOX-associated fluorescence, enhanced superoxide generation, and decreased sensitivity to DOX, findings consistent with altered redox cycling and oxidative processing of the drug. Re-expression of MB in MBKO cells restored resistance, whereas pharmacological modulation of MB function using carbon monoxide-releasing molecule-3 and *tert*-butoxycarbonyl-alanine reversed MB-dependent reductions in intracellular DOX accumulation. In contrast, aclarubicin, an anthracycline lacking the hydroquinone moiety required for efficient redox cycling, failed to reproduce MB-dependent effects. Analyses of four independent neoadjuvant BC cohorts further demonstrated that elevated MB expression was consistently associated with reduced probability of achieving pathological complete response following anthracycline-containing chemotherapy. Collectively, these findings identify MB as a previously unrecognized modulator of BC response to redox-active anthracyclines and support its potential utility as both a predictive biomarker and therapeutic target.

## 1. Introduction

Breast cancer (BC) remains the most frequently diagnosed malignancy among women worldwide and a leading cause of cancer-related mortality [1]. Anthracyclines such as doxorubicin (DOX) remain essential components of BC chemotherapy because of their potent efficacy, widespread availability, and continued integration into innovative drug delivery strategies [2]. However, their clinical efficacy is frequently compromised by intrinsic and acquired resistance mechanisms, including altered redox homeostasis, lysosomal sequestration, and drug efflux transporters [3,4,5,6,7], underscoring the need for reliable predictive biomarkers and a further understanding of the mechanisms governing anthracycline response and resistance. Myoglobin (MB), a heme-containing oxygen-binding globin classically expressed in cardiac and skeletal muscle, facilitates oxygen storage and diffusion and possesses a redox-active heme iron that enables participation in oxidative reactions [8]. MB is also expressed in several tumor types, including BC, where its presence has been associated with a more differentiated phenotype and a better prognosis [9,10]. Despite its well-established biochemical properties, the functional role of endogenous MB in cancer remains poorly understood. DOX bioactivation involves two interconnected redox modules: a cytotoxic pathway driven by NADPH-dependent reduction in DOX to semiquinone intermediates, and a redox cycling pathway that generates ROS (reviewed in Minotti et al., 2004) [11]. MB has emerged as an important regulator of DOX biotransformation, first in cell-free systems and later in cardiomyocytes, where DOX-induced ROS/H_2_O_2_ generation promotes formation of ferrylMB (MBIV), which subsequently catalyzes oxidative degradation of the DOX quinone/hydroquinone core into less toxic metabolites [12,13,14]. These observations prompted us to investigate whether endogenous MB modulates the response of BC cells to anthracyclines and thereby contributes to their therapeutic efficacy. This question is particularly relevant given the fundamentally distinct mechanisms underlying anthracycline toxicity in these cellular contexts. Whereas anthracycline-induced cardiotoxicity is strongly associated with ROS-mediated mitochondrial injury and oxidative stress, the antitumor activity of these agents primarily relies on DNA intercalation and topoisomerase IIα inhibition in proliferating cancer cells [4,15,16].

## 2. Materials and Methods

### 2.1. Cell Culture

Human BC cell lines (MCF7, SKBR3, MDA-MB-453, MDA-MB-231, MDA-MB-468) obtained from the American Type Culture Collection (ATCC, Manassas, VA, USA) were used in this study and were authenticated using the short tandem repeat profiling method. The Talen-recombinase-engineered MDA-MB-468 myoglobin knockout (MBKO) and its wild-type (WT) counterpart cells were a generous gift from Prof. Glen Kristiansen (Institute of Pathology, University Hospital Bonn, Germany). MCF7, SKBR3, MDA-MB-231 were cultured in Roswell Park Memorial Institute 1640 Medium (RPMI 1640, Gibco, Thermo Fisher Scientific, Waltham, MA, USA; Cat. No. 21875-034) MDA-MB-361, MDA-MB-453 and MDA-MB-468 were cultured in Dulbecco’s modified Eagle Medium (DMEM, Gibco, Thermo Fisher Scientific, Waltham, MA, USA; Cat. No. 41965-039) supplemented with 10% fetal bovine serum (FBS, Gibco, Thermo Fisher Scientific, Waltham, MA, USA; Cat. No. 16000), MDA-MB-468 MB (MB KO/WT) cells were cultured in DMEM high-glucose (Thermo Fisher Scientific, Waltham, MA, USA; Cat. No. 11965092) supplemented with 10% FBS and 1% penicillin/streptomycin (EuroClone, Pero, Italy; Cat. No. ECB3001D) and 1% GlutaMax (Thermo Fischer Scientific, Waltham, MA, USA; Cat. No. 35050061). Unless otherwise stated, cell lines were kept at 37 °C in humidified air with 5% CO_2_ and 21% O_2_.

### 2.2. Oxygen Deprivation

Experimental variants based on O_2_ deprivation were carried out in hypoxic glove-box chambers (InVivO_2_ 400, Ruskin Technologies, Bridgend, UK) maintained at 37 °C with 5% CO_2_. O_2_ concentration was continuously monitored using an internal O_2_ sensor. Hereafter, severe oxygen deprivation (0.2% O_2_) will be referred to as hypoxia. Cells were transferred to the hypoxia chamber 24 h after seeding and incubated for an additional 72 h, allowing the establishment of a stable hypoxic phenotype and sustained adaptation to low-oxygen conditions. All experiments carried out under hypoxia were performed in parallel with normoxic (21% O_2_) controls. Unless otherwise indicated, cells were analyzed immediately following completion of the 72 h normoxic or hypoxic exposure period.

### 2.3. Transient Transfection

To restore MB expression in MDA-MB-468 MBKO cells, 1 × 10^6^ cells were seeded in 6-well plates and transiently transfected 24 h later with 1 µg of the pmCherry-C1 vector encoding human myoglobin (*mCherryMB*), generated as previously described by Aboouf et al. [17], using Lipofectamine^®^ 2000 (3 µL) (Invitrogen, Thermo Fisher Scientific, Waltham, MA, USA; Cat. No. 11668019). As a control, the corresponding empty pmCherry-C1 vector (*mCherry*) was used. After 24 h, the culture medium was replaced, and cells were subjected to the indicated treatments under normoxic or hypoxic conditions as described above.

### 2.4. Drugs and Reagents

The following substances were added to the cell media: doxorubicin hydrochloride (DOX, Sigma-Aldrich, St. Louis, MO, USA; Cat. No. D1515); aclarubicin (ACLA, Santa Cruz Biotechnology, Dallas, TX, USA; Cat. No. sc-200160); carbon monoxide-releasing molecule 3 (CORM-3, Sigma–Aldrich, Cat. No. SML0496), used to generate carbon monoxide, which binds to the MB heme iron [18]; *tert*-Butoxycarbonyl-alanine (*t*-Boc-Ala, Sigma–Aldrich, St. Louis, MO, USA; Cat. No. 458457), used as a pharmacological inhibitor reported to interfere with interactions between DOX and the MB heme pocket [12] and chloroquine (cQ, Sigma–Aldrich, St. Louis, MO, USA; Cat. No. C6628). Stock solutions of DOX and ACLA were prepared in ultrapure sterile water and kept in the dark at 4 °C for up to two weeks. *t*-Boc-ala stock and CORM-3 solutions were prepared in ultrapure sterile water and kept at −20 °C. CQ stock solution was prepared in sterile phosphate-buffered saline (PBS; EuroClone, Pero, Italy; Cat. No. ECB4053). Stock preparations were further diluted in culture media immediately prior to being added to cells. During the whole incubation time, the cells were protected from light.

### 2.5. Cytotoxicity Assays

DOX IC_50_ was determined in the panel of BC cell lines by the sulforhodamine B (SRB) assay, as previously described [19]. The 3-(4,5-dimethylthiazol-2-yl)-2,5-diphenyltetrazolium bromide (MTT, Sigma–Aldrich, St. Louis, MO, USA; Cat. No. M5655) assay was used to evaluate the cytotoxic effect of DOX/ACLA in MDA-MB-468 cells. Briefly, cells were treated with different concentrations of DOX and incubated for 72 h under normoxia/hypoxia. After treatment, MTT solution was added to the cell medium to a final concentration of 0.5 mg/mL, and the plates were incubated for 1 h at 37 °C. Subsequently, the medium was removed, and cells were lysed by adding an equal volume of dimethyl sulfoxide (DMSO, Sigma-Aldrich, St. Louis, MO, USA; Cat. No. D8418). Optical density was measured at 560/670 nm using a Multiskan RC Microplate Photometer (Thermo Labsystems, Vantaa, Finland). To validate the results obtained by the MTT assay, we used the trypan blue exclusion assay to determine the number of viable cells following treatment of MDA-MB-468 (WT/MBKO) cells with DOX.

### 2.6. LAMP1-Positive Lysosome Analysis

To visualize lysosomes, cells were seeded on coverslips (2.9 × 10^4^ cells/well in 24-well plates), incubated overnight, and transduced with 5 µL of CellLight™ Lysosomes-GFP, BacMam 2.0 (Thermo Fisher Scientific, Waltham, MA, USA; Cat. No. C10507) according to the manufacturer’s instructions. After 6 h, the medium was replaced, and cells were incubated under normoxic or hypoxic conditions for 72 h. For confocal microscopy analysis, following treatment, cell monolayers were washed twice with PBS, fixed in 4% paraformaldehyde, and permeabilized with 0.1% Triton X-100 (Sigma-Aldrich, St. Louis, MO, USA; Cat. No. X100) in PBS. Cell nuclei were counterstained with 4′,6-diamidino-2-phenylindole (DAPI; Thermo Fisher Scientific, Waltham, MA, USA; Cat. No. D3571). Lysosomes and *mCherry*-tagged MB were visualized using a Leica SP8 inverted confocal microscope (Leica Microsystems, Wetzlar, Germany). For flow cytometry analysis (FACS), cells transduced with CellLight™ Lysosomes-GFP, BacMam 2.0 were washed with PBS, dissociated using trypsin–EDTA, fixed in 4% formaldehyde, and resuspended in PBS. Approximately 30,000 cells were analyzed per sample using a Gallios flow cytometer (Beckman Coulter, Brea, CA, USA), and data were processed with FlowJo software v10.10 (BD Biosciences, Ashland, OR, USA). GFP fluorescence was detected in the FITC channel (488 nm excitation, 530/30 nm emission) as a measure of LAMP1-positive lysosomal accumulation. Results are expressed as the geometric mean fluorescence intensity (X-GMean).

### 2.7. Quantification of Intracellular DOX Fluorescence by Flow Cytometry

DOX exhibits intrinsic fluorescence, which allows fluorescence-based assessment of intracellular DOX-associated signal by flow cytometry and confocal microscopy [20]. Following treatment with DOX and selected inhibitors, cells were washed with PBS, dissociated using trypsin–EDTA, fixed in 4% formaldehyde, and resuspended in PBS. Approximately 30,000 cells per sample were analyzed using a Gallios flow cytometer, and data were processed with FlowJo software. DOX fluorescence was detected in the PE channel (488 nm excitation, 575/26 nm emission). Results are presented as X-GMean after subtraction of background fluorescence measured in untreated control cells.

### 2.8. Measurement of Superoxide Production

Superoxide (O_2_•^−^) production was assessed by electron paramagnetic resonance (EPR) as previously described [21], with minor modifications. After treatment, cells were immediately washed twice with Krebs–HEPES buffer (pH 7.35, 99 mM NaCl, 4.69 mM KCl, 25 mM NaHCO_3_, 1.03 mM KH_2_PO_4_, 5.6 mM D-glucose, 20 mM Na-HEPES, 2.5 mM CaCl_2_, and 1.2 mM MgSO_4_) and were then incubated in the same buffer supplemented with 25 µM desferoxamine (Sigma–Aldrich, St. Louis, MO, USA; Cat. No. D9533), 5 µM diethyldithiocarbamate (Sigma–Aldrich, St. Louis, MO, USA; Cat. No. D3506), and 100 µM of the spin probe 1-hydroxy-3-methoxycarbonyl-2,2,5,5-tetramethylpyrrolidine (Enzo Life Sciences, Farmingdale, NY, USA; Cat. No. ALX-430-117) for 20 min on ice. Following incubation, samples were rapidly frozen in liquid nitrogen. For measurement, samples were thawed on ice, and EPR measurements were performed at 37 °C for 10 min using 10 scans on an eScan EPR spectrometer (Bruker BioSpin GmbH, Rheinstetten, Germany) equipped with temperature and gas controller (Noxygen Science Transfer & Diagnostics GmbH, Elzach, Germany). Data acquisition was performed using a center field of 3459–3466 G and a sweep width of 10 G. All other parameters were determined automatically by the autotune setting, resulting in a microwave power of 23.89 mW, a frequency of 9.7690 GHz, and a modulation amplitude of 2.93 G. O_2_•^−^ production rates were determined by linear regression and normalized to total cellular protein content.

### 2.9. Statistical Analysis

Statistical analyses for in vitro experiments were performed using GraphPad Prism 5.01 (GraphPad Software, San Diego, CA, USA). Differences between groups were evaluated using paired Student’s *t*-tests. Correlations between DOX IC_50_ values and gene expression levels were assessed using Spearman’s rank correlation analysis. *p*-values < 0.05 were considered statistically significant.

### 2.10. Public Breast Cancer Cohort Analyses

Publicly available BC transcriptomic datasets were obtained from the Gene Expression Omnibus (GEO), including GSE25055, GSE25065, GSE20194, and GSE41998. All cohorts included patients treated with anthracycline-containing neoadjuvant chemotherapy regimens with available pathological response annotations. Patients were classified according to the original dataset annotations as having achieved pathological complete response (pCR; no residual invasive cancer detected at surgery following neoadjuvant therapy) or residual disease (RD; persistent invasive cancer at surgery). Normalized bulk tumor gene expression matrices and corresponding clinical annotations were downloaded using the GEOquery package in R. *MB* expression values and clinicopathological annotations were extracted from each dataset. Associations between *MB* expression and pCR were evaluated using logistic regression models with pCR as the dependent variable and MB expression as the independent variable. Continuous *MB* expression values were used for primary analyses. Secondary dichotomous analyses were performed using median-based stratification into *MB*-high and *MB*-low groups within each cohort. Odds ratios (ORs) and 95% confidence intervals (95% CIs) were calculated from the model coefficients. Fixed-effect meta-analysis was performed using inverse variance weighting of cohort-specific log odds ratios. Tumor hypoxia was estimated using a reduced Buffa-derived hypoxia score, based on a previously validated transcriptomic hypoxia signature in BC [22,23]. The score was calculated as the mean expression of 10 canonical hypoxia-responsive genes (*VEGFA*, *SLC2A1*, *PGAM1*, *LDHA*, *ENO1*, *TPI1*, *NDRG1*, *ALDOA*, *ADM*, and *CA9*), which represent core components of the Buffa hypoxia metagene and include several of its highest-ranked and most prognostic hypoxia-associated genes [22]. Associations between *MB* expression and tumor hypoxia were assessed using Spearman correlation analysis. To further investigate whether *MB*-associated chemotherapy response was related to global hypoxia-associated transcriptional programs, exploratory pooled analyses were performed using this hypoxia score. For these analyses, *MB* expression and Buffa hypoxia scores were dichotomized using median-based stratification across the pooled dataset, generating *MB*-high/*MB*-low and hypoxia-high/hypoxia-low groups. pCR rates were subsequently compared across combined biological subgroups. Logistic regression analyses were performed to assess the independent and combined associations of *MB* expression and hypoxia-defined subgrouping with pCR, including an *MB*-hypoxia interaction term. All statistical analyses were performed in R version 4.6.0 (R Foundation for Statistical Computing, Vienna, Austria) using the tidyverse, ggplot2, broom, survival, survminer, GEOquery, and forestplot packages. All statistical tests were two-sided, and *p*-values < 0.05 were considered statistically significant.

## 3. Results

### 3.1. Myoglobin Mediates Hypoxia-Specific DOX Resistance in MDA-MB-468 Cells

**Figure 1:**
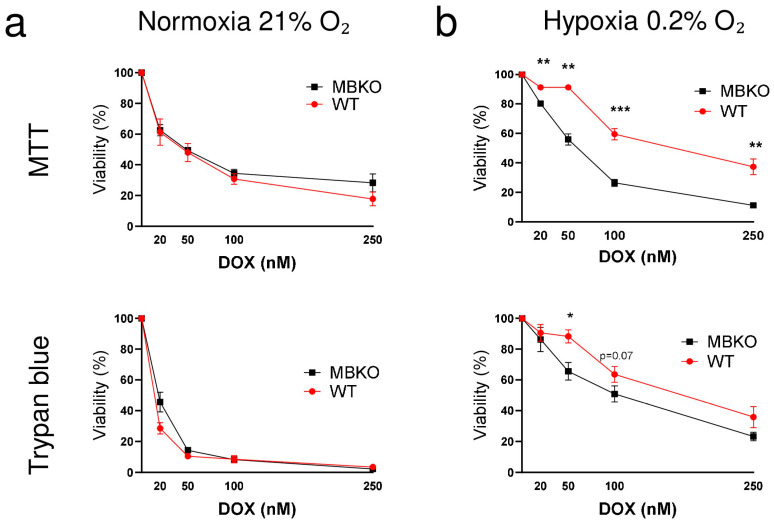
Myoglobin-dependent differences in doxorubicin sensitivity in MDA-MB-468 cells are revealed under hypoxia. Effects of increasing concentrations of doxorubicin (DOX; 20, 50, 100, and 250 nM) on the viability of MDA-MB-468 wild-type (WT) and myoglobin knockout (MBKO) cells were assessed after 72 h under normoxic (21% O_2_; (**a**)) and hypoxic (0.2% O_2_; (**b**)) conditions. Cell viability was determined by MTT assay (**upper** panels) and the trypan blue exclusion assay (**lower** panels). Data are expressed as a percentage of untreated controls and presented as mean ± SEM from four independent experiments (n = 4). Statistical significance was determined using a paired Student’s *t*-test (* *p* < 0.05, ** *p* < 0.01, *** *p* < 0.001).

DOX sensitivity was initially evaluated across a panel of BC cell lines representing distinct molecular subtypes. Sulforhodamine B assays demonstrated marked variability in response, with MDA-MB-468 cells showing the highest sensitivity, whereas MCF7 and SKBR3 cells were the most resistant (Figure S1a). RT–qPCR analyses revealed heterogeneous expression of *MB* and the canonical DOX resistance-associated transporters *ABCC2* and *ABCG2*, with *ABCG2* showing the strongest association with reduced DOX sensitivity (Figure S1b,c). Given that anthracyclines remain a central component of triple-negative breast cancer (TNBC) chemotherapy [24], we selected the MB-expressing TNBC cell line, MDA-MB-468, and its TALEN-engineered MB knockout derivative (MBKO) (characterized by Armbruster et al., [25]) to investigate the role of MB in DOX response. MB depletion was confirmed by Western blot (Figure S2). Considering the oxygen-dependent properties of MB, DOX sensitivity was evaluated under normoxic (21% O_2_) and hypoxic (0.2% O_2_) conditions, the latter being expected to promote extensive MB deoxygenation [26]. Under normoxia, MDA-MB-468 WT and MBKO cells exhibited comparable dose-dependent responses to DOX, as assessed by MTT and trypan blue exclusion assays (Figure 1a). In contrast, under hypoxia, MBKO cells exhibited significantly increased sensitivity to DOX compared with WT cells across all tested concentrations in the MTT assay, with similar trends observed using the trypan blue exclusion assay (Figure 1b). Based on these results, subsequent analyses were performed using the MTT assay as a proxy for cell viability. To further establish a causal role for MB in modulating DOX response, MB was re-expressed in MBKO cells using an *mCherryMB* construct, with successful re-expression confirmed by RT–qPCR (Figure S3a). MB re-expression significantly increased cell viability under hypoxic conditions compared with control-transfected cells (Figure S3b).

### 3.2. Myoglobin Regulates Doxorubicin Redox Cycling, Intracellular Accumulation, and Cytotoxicity Under Hypoxia

**Figure 2:**
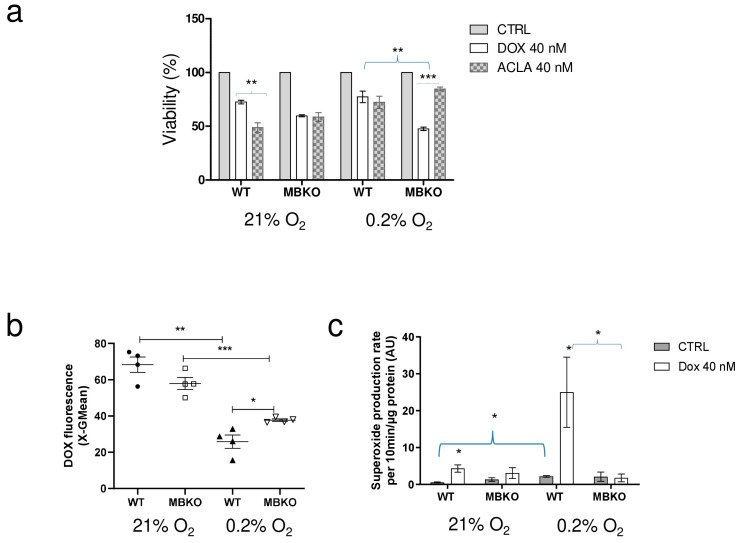
Myoglobin uncouples doxorubicin-induced superoxide generation from cytotoxicity while modulating intracellular doxorubicin accumulation under hypoxia. MDA-MB-468 wild-type (WT) and myoglobin knockout (MBKO) were treated with doxorubicin (DOX, 40 nM) and aclarubicin (ACLA, 40 nM) for 72 h under normoxic (21% O_2_) or hypoxic (0.2% O_2_) conditions. Cell viability was assessed by the MTT assay. Data are expressed as a percentage of untreated controls (**a**); Intracellular DOX fluorescence was quantified by flow cytometry based on DOX intrinsic fluorescence and expressed as the geometric mean fluorescence intensity (X-Gmean), with each dot corresponding to one independent experiment; different symbol shapes are used to distinguish the experimental groups shown on the x-axis. (**b**); Superoxide production rate was quantified by electron paramagnetic resonance spectroscopy (**c**). Results are presented as a mean ± SEM from a minimum of three independent experiments. Statistical significance was determined using a paired Student’s *t*-test (* *p* < 0.05, ** *p* < 0.01, *** *p* < 0.001).

We next used a single, biologically active concentration of DOX (40 nM) to enable direct comparison of cellular responses under normoxic and hypoxic conditions. Consistent with our dose–response analyses, under hypoxia (0.2% O_2_), MBKO cells displayed significantly reduced viability compared with WT cells (WT ~75% vs. MBKO ~50%, *p* < 0.01), supporting a protective role of MB under low-oxygen conditions (Figure 2a). To determine whether this effect depends on the redox properties of the drug, we tested the anthracycline analog aclarubicin (ACLA; 40 nM), which lacks the hydroquinone moiety in its chromophore (Figure S4b) and is therefore less susceptible to redox cycling and oxidative degradation by MB [12]. Under normoxia, ACLA reduced cell viability similarly in WT and MBKO cells. However, under hypoxic conditions, ACLA did not reproduce the MB-dependent differences in cytotoxicity observed with DOX. Notably, MBKO cells were significantly more resistant to ACLA compared with DOX (~1.8-fold increase in viability, *p* < 0.001), indicating a distinct drug response (Figure 2a). To determine whether MB influences intracellular DOX accumulation, DOX-associated fluorescence was quantified by FACS in WT and MBKO MDA-MB-468 cells following 72 h of exposure to normoxic or hypoxic conditions. Under hypoxia, both WT and MBKO cells exhibited significantly reduced DOX fluorescence compared with their respective normoxic controls (WT: ~3-fold decrease, *p* < 0.01; MBKO: ~1.5-fold decrease, *p* < 0.001), indicating an overall reduction in intracellular DOX levels in low oxygen conditions. Notably, this effect was more pronounced in WT cells, which displayed significantly lower DOX fluorescence than MBKO cells under hypoxia (~1.4-fold difference, *p* < 0.05) (Figure 2b).

Next, we investigated superoxide (O_2_•^−^) formation by EPR spectroscopy in WT and MBKO MDA-MB-468 cells following 72 h of DOX treatment under normoxic or hypoxic conditions. Under basal conditions (CTRL), no significant differences in O_2_•^−^ levels were observed between WT and MBKO cells under either oxygen condition. However, hypoxia alone significantly increased O_2_•^−^ levels in WT cells compared with normoxic WT CTRL cells (*p* < 0.05), whereas no such effect was observed in MBKO cells, suggesting that MB contributes to hypoxia-induced ROS generation. DOX treatment markedly increased O_2_•^−^ formation in WT cells under both oxygen conditions, resulting in an approximately 9-fold increase in normoxia and a 12-fold increase in hypoxia relative to their respective CTRL groups (both *p* < 0.05). In contrast, DOX did not significantly enhance O_2_•^−^ production in MBKO cells. Consequently, under hypoxia, DOX-treated WT cells exhibited approximately 14-fold higher O_2_•^−^ levels than DOX-treated MBKO cells (*p* < 0.05) (Figure 2c). These data indicate that MB facilitates DOX-driven redox cycling, leading to enhanced O_2_•^−^ generation under hypoxia, yet this increase does not translate into greater cytotoxicity. Consistent with these findings, re-expression of MB also significantly affected the cellular response to DOX. Upon treatment with 40 nM DOX, MB-reconstituted cells (*mCherryMB*) exhibited increased viability under hypoxia compared with control-transfected cells (*mCherry*) (*p* < 0.01), mirroring the protective phenotype observed in WT cells. In contrast, no substantial differences were observed under normoxic conditions (Figure S5a). Re-expression of MB recapitulated the WT phenotype, resulting in reduced intracellular DOX fluorescence under hypoxia (*p* < 0.05) (Figure S5b), thereby confirming a causal role of MB in modulating DOX accumulation.

### 3.3. Myoglobin Promotes Hypoxia-Dependent Increases in Cellular Granularity and Lysosomal Accumulation

**Figure 3:**
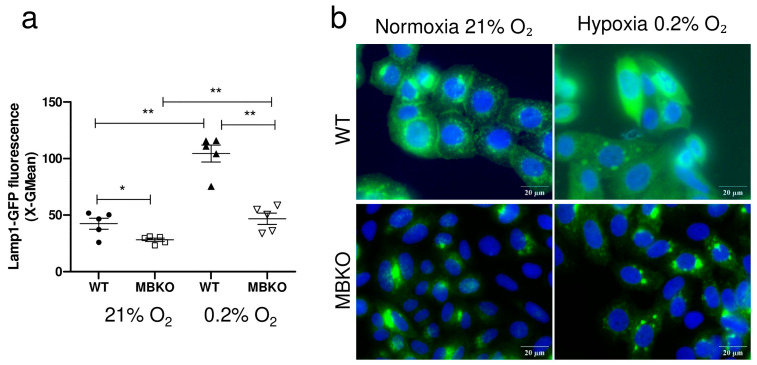
Myoglobin-expressing MDA-MB-468 cells display increased LAMP1-positive vesicles. MDA-MB-468 wild-type (WT) and myoglobin knockout (MBKO) cells were incubated for 72 h under normoxic (21% O_2_) or hypoxic (0.2% O_2_) conditions with BacMam expression vectors encoding GFP fused to the lysosomal targeting sequence of LAMP1 (lysosomal-associated membrane protein 1). LAMP1-GFP expression was quantified by flow cytometry and reported as geometric mean fluorescence intensity (X-GMean). Each data point represents an independent experiment; different symbol shapes are used to distinguish the experimental groups shown on the x-axis. Data are presented as mean ± SEM (n = 5). Statistical significance was assessed using a paired Student’s *t*-test (* *p* < 0.05, ** *p* < 0.01). The horizontal line indicates the group mean (**a**). LAMP1 is shown in green, and nuclei were counterstained with DAPI (blue). Representative images were acquired using a 100× oil immersion EC Plan-Neofluar objective (NA 1.30; Carl Zeiss AG, Oberkochen, Germany). Scale bar = 20 μm (**b**).

FACS analysis revealed that WT MDA-MB-468 cells exhibited significantly higher side-scatter (SSC-H) values after 72 h of hypoxia than MBKO cells (Figure S6a). This observation was further confirmed following MB re-expression, as *mCherryMB* cells displayed significantly higher SSC-H levels than empty vector controls (*mCherry*) (Figure S6b). Because increased SSC-H is commonly associated with increased intracellular complexity, these findings indicate that MB expression is associated with increased intracellular granularity under hypoxic conditions. We therefore examined lysosomal accumulation using LAMP1-GFP labeling. Under normoxia, WT cells displayed significantly higher LAMP1-GFP fluorescence than MBKO cells (~1.5-fold, *p* < 0.05). Hypoxia further increased LAMP1-GFP fluorescence in WT cells (~2.5-fold relative to normoxic WT, *p* < 0.01), whereas this response was markedly attenuated in MBKO cells, which exhibited ~2-fold lower fluorescence than WT cells under hypoxia (*p* < 0.01) (Figure 3a,b).

### 3.4. Pharmacological Modulation of Myoglobin and Lysosomal Function Alters Doxorubicin Accumulation Under Hypoxia

**Figure 4:**
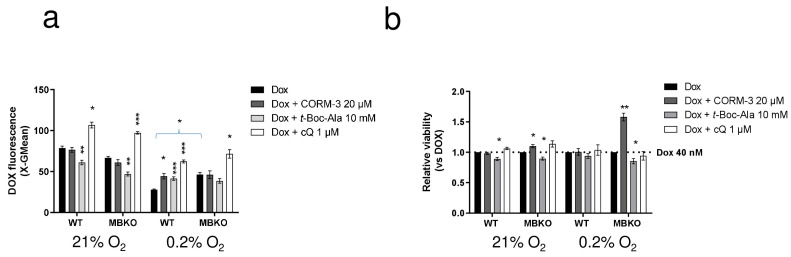
Modulation of doxorubicin fluorescence and cytotoxicity by redox- and lysosome-targeting agents. MDA-MB-468 wild-type (WT) and myoglobin knockout (MBKO) cells were treated for 72 h with doxorubicin (DOX, 40 nM) alone or in combination with carbon monoxide-releasing molecule-3 (CORM-3, 20 µM), *tert*-Butoxycarbonyl-alanine (*t*-Boc-Ala, 10 mM), or chloroquine (cQ, 1 µM) under normoxic (21% O_2_) and hypoxic (0.2% O_2_). Intracellular DOX fluorescence was quantified by flow cytometry and expressed as the geometric mean fluorescence intensity (X-Gmean) (**a**). Cell viability was assessed by the MTT assay. Data are presented as ratios relative to the DOX treatment alone (**b**). Values represent mean ± SEM from a minimum of three independent experiments. Statistical significance was determined using a paired Student’s *t*-test (* *p* < 0.05, ** *p* < 0.01, *** *p* < 0.001).

To investigate the mechanisms underlying MB-dependent modulation of DOX handling, we assessed the impact of pharmacological modulators of MB activity and lysosomal function on intracellular DOX accumulation and cytotoxicity. Treatment with CORM-3 had no significant effect under normoxia in either WT or MBKO cells (not significant (n.s.) vs. DOX). In contrast, under hypoxia, CORM-3 significantly increased DOX fluorescence in WT cells (~1.5-fold vs. DOX, *p* < 0.05), restoring levels comparable to those in MBKO cells (WT vs. MBKO: n.s.). These findings suggest that CO binding interferes with MB redox activity, thereby preventing MB-mediated DOX degradation. Similarly, inhibition of the MB–DOX interaction using *t*-Boc-Ala markedly increased DOX fluorescence in hypoxic WT cells (~1.5-fold vs. DOX alone, *p* < 0.01), while exerting only minor effects under normoxia. Importantly, *t*-Boc-Ala abolished the difference between WT and MBKO cells under hypoxia (WT vs. MBKO: n.s.), further supporting a direct role for MB in DOX processing. Disruption of lysosomal function with chloroquine (cQ) markedly increased DOX fluorescence in both WT and MBKO cells under normoxic and hypoxic conditions (Figure 4a). CQ increased DOX fluorescence by ~1.5–2-fold across all conditions (*p* < 0.01 vs. DOX alone), indicating accumulation of intact intracellular DOX following lysosomal inhibition. Notably, cQ abolished the difference in DOX fluorescence between WT and MBKO cells under hypoxia (WT vs. MBKO: n.s.), suggesting that MB-dependent DOX clearance requires functional lysosomal activity. However, the magnitude of cQ-induced DOX accumulation was comparable across genotypes and oxygen conditions, indicating that this effect is largely independent of MB expression and oxygen tension. Under hypoxia, modulation of MB activity also affected cellular responses to DOX (Figure 4b). CORM-3 treatment significantly increased viability in MBKO cells (~1.5-fold vs. DOX alone, *p* < 0.01), suggesting additional effects of the compound beyond direct MB inhibition. In contrast, *t*-Boc-Ala modestly reduced viability (*p* < 0.05) (Figure 4b). A mild reduction in viability was also observed in MBKO cells treated with *t*-Boc-Ala under normoxia, indicating a potential off-target toxic effect. WT cells showed no significant changes in viability following pharmacological treatments. Overall, these findings indicate that pharmacological modulation of MB-related pathways strongly affects intracellular DOX accumulation while exerting comparatively limited effects on short-term cytotoxicity.

### 3.5. High Myoglobin Expression Is Associated with Reduced Response to Anthracycline-Containing Chemotherapy

*MB* expression was evaluated in four independent BC cohorts treated with neoadjuvant chemotherapy, including GSE25055 (n = 156), GSE25065 (n = 100), GSE20194 (n = 140), and GSE41998 (n = 140). Clinical, molecular, and treatment characteristics of the analyzed cohorts are summarized in Table 1.

**Figure 5:**
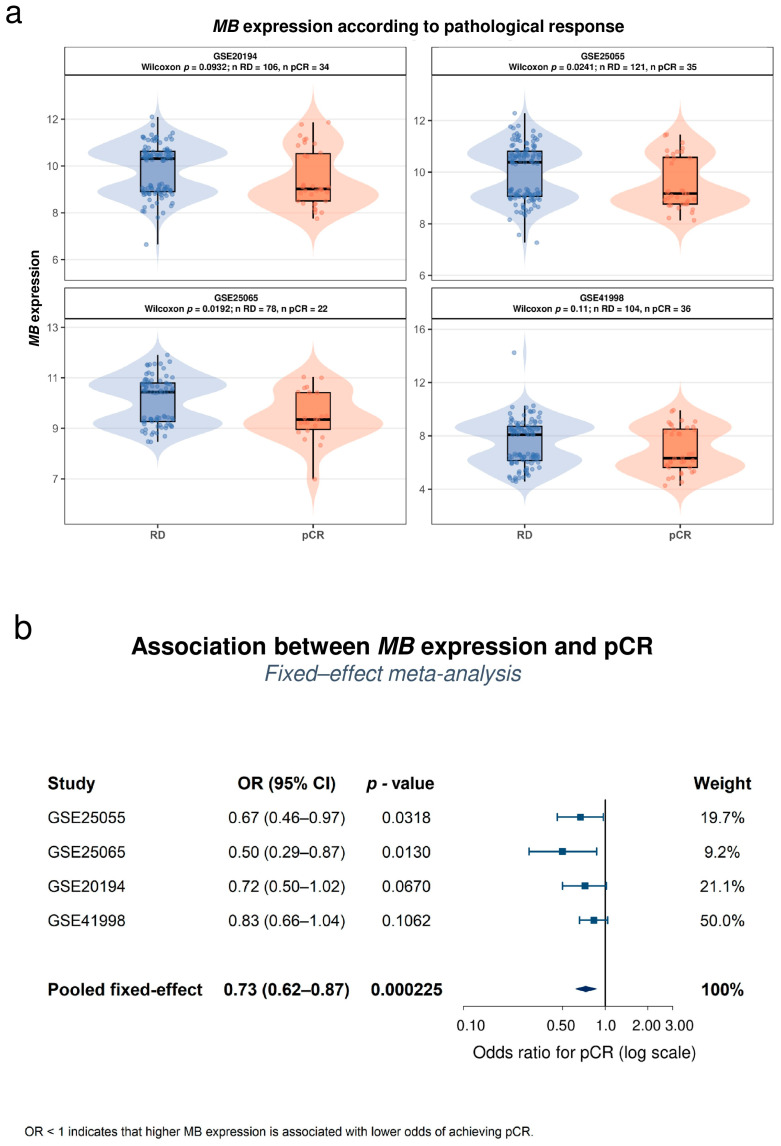
High *myoglobin* expression is associated with reduced pathological complete response across breast cancer neoadjuvant chemotherapy cohorts. Violin/boxplots showing *MB* expression levels according to pathological response status in GSE25055, GSE25065, GSE20194, and GSE41998 cohorts (**a**). Forest plot showing cohort-specific and pooled odds ratios (ORs) for pathological complete response (pCR) according to continuous *MB* expression. ORs below 1 indicate lower odds of achieving pCR with higher MB expression. Odds ratios and 95% confidence intervals (95% CIs) were derived from logistic regression analyses. Fixed-effect meta-analysis was performed using inverse variance weighting (**b**).

pCR rates ranged from 22.0% to 25.7% across cohorts. Tumors with RD consistently demonstrated higher MB expression than tumors achieving pCR (Figure 5a). Continuous logistic regression analyses demonstrated concordant negative associations between *MB* expression and pCR across cohorts. A mixed-effect pooled meta-analysis confirmed that higher *MB* expression was significantly associated with a reduced probability of achieving pCR following neoadjuvant chemotherapy (pooled OR 0.73, 95% CI 0.62–0.87, *p* = 0.000225; Figure 5b).

**Figure 6:**
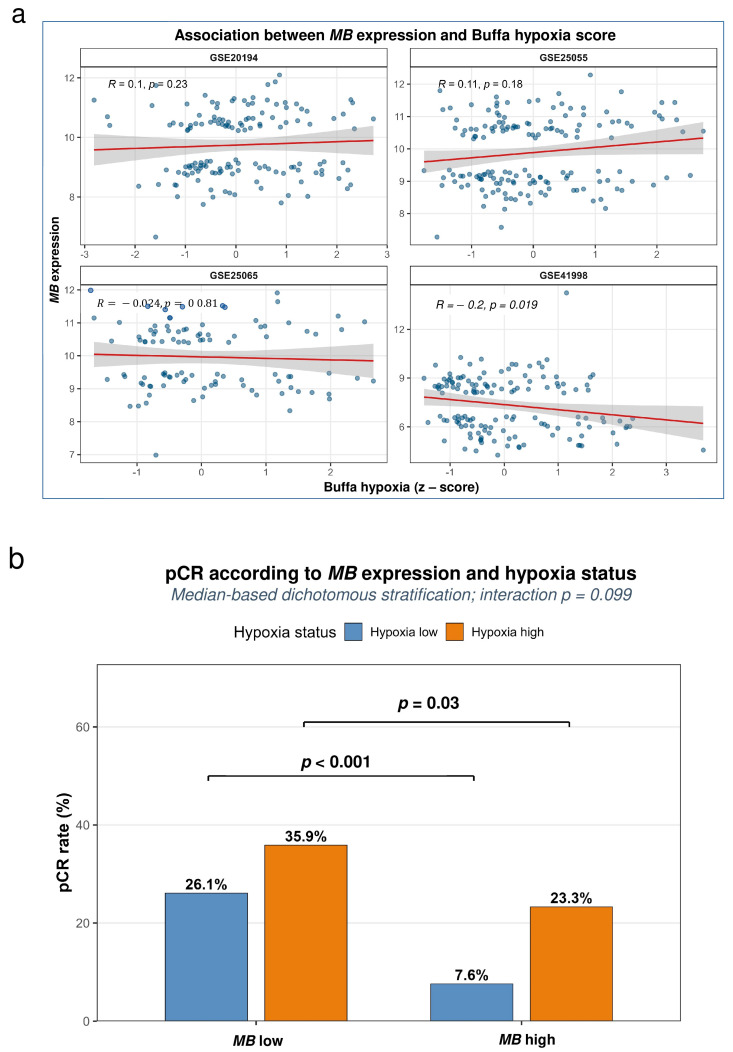
*Myoglobin*-associated pathological response is not explained by global tumor hypoxia signatures. Scatterplots showing the relationship between *MB* expression and the Buffa hypoxia score across GSE25055, GSE25065, GSE20194, and GSE41998 breast cancer (BC) neoadjuvant chemotherapy cohorts. Red lines indicate linear regression trends with shaded 95% confidence intervals. Spearman correlation coefficients (R) and corresponding *p*-values are shown for each cohort (**a**). Tumors with available *MB* expression, Buffa hypoxia score, and pathological complete response (pCR) data were included (n = 536). Median-based dichotomization was used to define *MB*-high/*MB*-low and hypoxia-high/hypoxia-low groups. Logistic regression analysis demonstrated significant independent effects of *MB* expression and hypoxia status on pCR, whereas the interaction between *MB* and hypoxia status showed a non-significant trend (interaction *p* = 0.099) (**b**).

Exploratory dichotomous analyses using median-based *MB* stratification demonstrated similar findings. *MB*-high tumors demonstrated lower odds of achieving pCR compared with *MB*-low tumors in GSE25055 (OR 0.43, 95% CI 0.20–0.95, *p* = 0.037), GSE25065 (OR 0.38, 95% CI 0.14–1.03, *p* = 0.058), GSE20194 (OR 0.38, 95% CI 0.17–0.86, *p* = 0.020), and GSE41998 (OR 0.64, 95% CI 0.30–1.37, *p* = 0.248). Fixed-effect pooled meta-analysis further demonstrated that *MB*-high tumors were significantly associated with a lower probability of pCR (pooled OR 0.46, 95% CI 0.30–0.70, *p* = 0.00026; Supplementary Figure S7). The association between *MB* expression and the Buffa hypoxia score was weak and inconsistent across the four cohorts. Weak positive, non-significant correlations were observed in GSE20194 (R = 0.10, *p* = 0.23) and GSE25055 (R = 0.11, *p* = 0.18), while no correlation was detected in GSE25065 (R = −0.024, *p* = 0.81). Although GSE41998 showed a statistically significant inverse correlation (R = −0.20, *p* = 0.019), the effect size was modest (Figure 6a). To further evaluate whether *MB*-associated chemotherapy response persisted across hypoxia-defined tumor groups, exploratory pooled analyses were performed across all four neoadjuvant BC cohorts. Only tumors with available *MB* expression, Buffa hypoxia score, and pCR annotations were included, resulting in a pooled dataset of 536 tumors. *MB* expression and Buffa hypoxia scores were dichotomized using median-based stratification, generating *MB*-high/*MB*-low and hypoxia-high/hypoxia-low groups. Combined *MB*/hypoxia stratification revealed marked differences in pCR rates across biological subgroups (Figure 6b). Within hypoxia-high tumors, *MB*-low cases had significantly higher pCR rates than *MB*-high cases (35.9% vs. 23.3%, *p* = 0.03). Similarly, within hypoxia-low tumors, *MB*-low tumors also exhibited substantially higher pCR rates than *MB*-high tumors (26.1% vs. 7.6%, *p* < 0.001).

Logistic regression analyses further demonstrated significant independent effects of both *MB* expression and hypoxia-defined subgrouping on pCR. *MB*-low tumors exhibited significantly higher odds of achieving pCR compared with *MB*-high tumors, independently of hypoxia grouping (*p* = 0.030). In contrast, the *MB* and hypoxia interaction term showed only a non-significant trend (*p* = 0.099), indicating that the association between *MB* expression and pCR was largely preserved across hypoxia-defined subgroups rather than being strictly dependent on global hypoxia status.

## 4. Discussion

In this study, we identify endogenous MB as a determinant of anthracycline responsiveness in BC cells. Our data indicate that MB is associated with reduced DOX fluorescence, enhanced redox activity, and attenuated DOX efficacy under hypoxia, supporting a model in which deoxyMB may promote redox-dependent oxidative processing of the drug and reduced intracellular DOX availability. Importantly, these experimental findings were complemented by analyses of four independent retrospective anthracycline-based neoadjuvant BC cohorts, in which elevated *MB* expression was consistently associated with lower rates of pCR following treatment, supporting a potential role for MB as a predictive biomarker of anthracycline response. Reduced DOX toxicity in MB-expressing hypoxic BC cells was paralleled by lower intracellular DOX-associated fluorescence and increased O_2_•^−^ generation. Because the intrinsic fluorescence of DOX depends on the preservation of its intact tetracyclic anthracycline scaffold [14], the concomitant reduction in DOX-associated fluorescence and increase in O_2_•^−^ generation are consistent with enhanced redox-dependent processing of the drug. Collectively, these findings support the notion that, under hypoxia, deoxyMB acquires functionally redox-active properties and redirects DOX biotransformation away from productive cytotoxic activity toward ROS-generating redox cycling. MB function critically depends on the ligand coordinated to the heme iron (e.g., O_2_ or CO) [27,28]. In this context, CORM-3, through CO release, is expected to shift MB toward the carboxymyoglobin (MBCO) state. Previous studies have demonstrated that CO readily binds intracellular MB under physiologically relevant hypoxic conditions, supporting MBCO formation in our experimental setting [18]. Occupancy of the heme iron by CO is expected to reduce MB availability for redox cycling and thereby limit the generation of ferrylMB intermediates implicated in anthracycline degradation [27]. Consistent with this model, CORM-3 selectively increased intracellular DOX fluorescence in hypoxic MB-expressing cells, supporting the preservation of structurally intact anthracycline. Similarly, *t*-Boc-Ala, which interferes with interactions between DOX and the MB heme pocket [12], increased intracellular DOX levels specifically under hypoxic conditions in MB-expressing cells. Although increased intracellular retention of intact DOX would be expected to enhance cytotoxic efficacy, the pharmacological data should be interpreted cautiously. CORM-3 reduced DOX toxicity in MB-deficient cells, indicating additional MB-independent effects on anthracycline response [29,30]. In contrast, *t*-Boc-Ala produced only a modest sensitizing effect across experimental conditions, potentially reflecting intrinsic toxicity or off-target activity of the compound itself. Therefore, these pharmacological experiments should be interpreted as supportive evidence consistent with the proposed mechanism and complementary to the genetic loss-of-function and rescue experiments. MB-expressing cells accumulated LAMP1-positive vesicles under hypoxia, consistent with previous studies linking deoxygenated or redox-active MB states to oxidative stress and lysosomal remodeling [25]. Interestingly, Montes et al. demonstrated that the His98Tyr MB mutation, which impairs MB–oxygen binding, also promotes O_2_•^−^ generation together with accumulation of LAMP1-positive lysosomal/autophagic structures [31]. Because DOX is known to accumulate within acidic lysosomal compartments [32], this phenotype could potentially influence intracellular drug availability. However, cQ-mediated disruption of lysosomal acidification altered intracellular DOX accumulation similarly in both MB-expressing and MB-deficient cells, suggesting that lysosomal function contributes broadly to DOX handling rather than specifically mediating the MB-dependent phenotype. Aclarubicin (ACLA), a DOX analog currently attracting renewed interest due to its lower cardiotoxicity and emerging therapeutic potential despite historically limited efficacy in BC [33,34,35], did not reproduce the hypoxia-dependent MB modulation observed with DOX. Interestingly, under hypoxia, MBKO cells remained significantly more resistant to ACLA than to DOX. This observation likely reflects the distinct redox properties of these anthracyclines. Because ACLA lacks the hydroquinone moiety required for efficient anthracycline redox cycling, MB ablation would be expected to preferentially affect the cytotoxic activity of DOX rather than ACLA, supporting the notion that MB primarily modulates the activity of redox-active anthracyclines.

Collectively, our findings support a model in which deoxyMB preferentially promotes oxidative turnover of redox-active anthracyclines through ferrylMB/peroxide-dependent redox mechanisms. The association between MB expression and reduced DOX sensitivity observed in vitro was paralleled by findings from independent neoadjuvant BC cohorts, where elevated *MB* expression was associated with a lower probability of achieving pCR following anthracycline-containing chemotherapy, supporting the potential translational relevance of our findings. Exploratory pooled analyses further showed that *MB*-low tumors exhibited higher pCR rates than *MB*-high tumors across both hypoxia-high and hypoxia-low subgroups. Although MB-dependent effects in vitro were primarily observed under hypoxia (0.2% O_2_), conditions favoring the predominance of deoxyMB, oxygen levels within solid tumors commonly range between 0.02% and 2% O_2_ [26,27], suggesting that the hypoxic conditions used in our study likely reflect physiologically relevant oxygenation states encountered in breast tumors more closely than conventional cell culture normoxia (21% O_2_). Notably, the relationship between *MB* expression and the Buffa hypoxia score was heterogeneous across the four cohorts, ranging from weak positive to modest negative correlations, with only one cohort reaching statistical significance. This observation is consistent with previous findings by Kristiansen et al., showing that MB expression in BC only partially overlaps with classical hypoxic tumor regions [36]. These findings indicate that the clinical association between *MB* and anthracycline response is not explained by global tumor hypoxia. Rather, they suggest that, within the physiologically low oxygen tensions encountered in solid tumors, transcriptomic hypoxia signatures reflect a more complex biological response than oxygen tension alone and, therefore, are not directly equivalent to the local oxygen environment that determines the biochemical state and activity of MB.

Despite the marked clinical and molecular heterogeneity within the four independent anthracycline-treated neoadjuvant BC cohorts, the inverse association between *MB* expression and pCR was consistently observed. Because MB expression is enriched in luminal BC, a subtype generally characterized by lower pCR rates following neoadjuvant chemotherapy [37], the influence of intrinsic molecular subtype cannot be completely excluded in these retrospective analyses, as subtype-stratified analyses were limited by statistical power. At the same time, despite decades of clinical use, no clinical, pathological, or molecular biomarker has been conclusively validated to identify patients who specifically benefit from anthracycline-containing chemotherapy [38]. In this context, our genetic loss-of-function and rescue experiments demonstrate that MB modulates cellular sensitivity to DOX under controlled experimental conditions, supporting a functional contribution of MB to anthracycline response rather than merely reflecting luminal tumor biology. Additional support for the biological relevance of MB in anthracycline response comes from murine mammary carcinoma studies reported by Aboouf et al., where anthracycline treatment selectively reduced metastatic burden in MB-deficient tumors [39]. MB expression in DOX-treated hypoxic cells shifted the intracellular redox balance toward enhanced O_2_•^−^ generation, a phenomenon that may have implications extending beyond altered anthracycline metabolism. Because superoxide signaling has been implicated in the regulation of BC stem-like cells [40], MB-dependent ROS remodeling could potentially contribute not only to reduced DOX efficacy but also to adaptive cellular states associated with tumor progression and therapy resistance.

The following limitations should be acknowledged. Our mechanistic analyses were performed primarily in vitro using a single TNBC model (MDA-MB-468), despite the substantial molecular and phenotypic heterogeneity of TNBC. In addition, although the consistent association between elevated *MB* expression and reduced pCR across independent cohorts supports the clinical relevance of our findings, the translational analyses were based on retrospective public datasets and require larger prospective studies with comprehensive subtype-specific analyses. Furthermore, although our data are consistent with a role for deoxyMB/ferrylMB-dependent modulation of DOX-associated fluorescence and redox activity, MB-mediated oxidative degradation of DOX was not directly demonstrated. In particular, reduced DOX fluorescence should be interpreted as an indirect readout of altered DOX-associated signal rather than a direct measurement of intact drug. Direct analytical approaches, such as HPLC or mass spectrometry, will be required to identify potential DOX metabolites and conclusively establish whether MB promotes DOX degradation in BC cells. Future studies using orthotopic and patient-derived tumor models will be important to determine whether MB can robustly predict anthracycline responsiveness and disease progression in vivo. In addition, while the present study primarily focused on cell viability and intracellular drug metabolism, it will be important to investigate whether MB-dependent redox remodeling influences stem cell–related phenotypes, cellular plasticity, or metastatic progression.

## 5. Conclusions

Overall, our findings identify MB as a previously unrecognized modulator of anthracycline response in BC cells. Under hypoxic conditions, deoxyMB promoted enhanced O_2_•^−^ generation, reduced DOX-associated fluorescence, and decreased cellular sensitivity to DOX, consistent with a model in which ferrylMB-dependent redox cycling contributes to altered intracellular DOX availability. The concordance between these in vitro findings and the consistent association of elevated *MB* expression with reduced pCR across four independent anthracycline-treated neoadjuvant BC cohorts provides a rationale for the prospective validation of MB as a candidate predictive biomarker of anthracycline response.

## Figures and Tables

**Table 1:** Clinical and molecular characteristics of breast cancer neoadjuvant chemotherapy cohorts.

Cohort	Regimen	Patients (n)	pCR n (%)	ER n (%)	PR n (%)	HER2 n (%)	TNBC n (%)	PAM50 Basal-Like n (%)
GSE25055	Taxane + FAC/FEC	156	35 (22.4%)	82 (52.6%)	67 (42.9%)	2 (1.3%)	64 (41.0%)	66 (42.3%)
GSE25065	Taxane + FAC/FEC	100	22 (22.0%)	59 (59.0%)	50 (50.0%)	1 (1.0%)	36 (36.0%)	40 (40.0%)
GSE20194	PTX + FAC	140	34 (24.3%)	75 (53.6%)	56 (40.0%)	30 (21.4%)	40 (28.6%)	NA
GSE41998	AC → TXA/PTX	140	36 (25.7%)	47 (33.6%)	48 (34.3%)	13 (9.3%)	77 (55.0%)	NA

Values are presented as number (n) and percentage (%). Abbreviations: pCR, pathological complete response; ER, estrogen receptor; PR, progesterone receptor; HER2, human epidermal growth factor receptor 2; TNBC, triple-negative breast cancer; PAM50 basal-like, basal-like intrinsic molecular subtype defined by PAM50 gene expression profiling; PTX, paclitaxel; FAC, fluorouracil, doxorubicin, and cyclophosphamide; FEC, fluorouracil, epirubicin, and cyclophosphamide; AC, doxorubicin and cyclophosphamide; IXA, ixabepilone; NA, not available.

## Data Availability

The original contributions presented in this study are included in the article/Supplementary Material. Further inquiries can be directed to the corresponding authors.

## Supplementary Materials

The following supporting information can be downloaded at https://www.mdpi.com/article/10.3390/biom16071055/s1, Figure S1: DOX IC_50_ and basal expression of myoglobin in breast cancer cell lines. IC_50_ values of DOX (µM) evaluated in breast cancer cell lines (MCF7, SKBR3, MDA-MB-453,-468,-231) after 72 h of incubation with different doses of DOX; toxicity was measured by sulforhodamine B assay. Each dot in the scatter plot represents an independent experiment. Lines show the mean IC_50_ doses derived from three independent experiments. All the experiments were performed in normoxia (21% O_2_) (a). mRNA expression of myoglobin (*MB*), ATP Binding Cassette Subfamily C Member 2 (*ABCC2*) and ATP Binding Cassette Subfamily G Member 2 (*ABCG2*) in breast cancer cell lines (MCF7, SKBR3, MDA-MB-453,-468,-231) was evaluated by qPCR. Results are presented as the mean ± SEM of three independent experiments. 18S was used as housekeeping gene. All experiments were performed in normoxia (21% O_2_) (b). Correlation analysis between doxorubicin (DOX) IC_50_ values (µM, x-axis) and mRNA expression levels of *MB*, *ABCC2*, and *ABCG2* (y-axis, expressed as 2^^-^DCt) across a panel of breast cancer cell lines (MCF7, SKBR3, MDA-MB-453, MDA-MB-468, and MDA-MB-231). Associations were assessed using Spearman’s rank correlation coefficient (r) (c); Figure S2: Myoglobin expression in MDA-MB-468 cell line. Western blot analysis of myoglobin (MB) in wild-type (WT) and MB knockout (MBKO) MDA-MB-468 cell lines. Data are representative of three independent cell passages. Beta actin (BACTIN) was used as a loading control; Figure S3: Myoglobin re-expression in MBKO cells confers hypoxia-dependent protection against doxorubicin-induced cytotoxicity. Expression of myoglobin (MB) evaluated by qPCR analysis of myoglobin (MB) mRNA in MDA-MB-468 cell line transfected with empty (*mCherry*) and MB containing (mCherryMB) vectors. 18S was used as housekeeping gene. *** *p* < 0.001, by paired Student’s *t*-test (a). Effects of various concentrations of doxorubicin (DOX) (10, 20 and 40 nM) on MDA-MB-468 MBKO cells (*mCherry*/*mCherryMB*) viability measured after 72 h of incubation in normoxia (21% O_2_, left) and hypoxia (0.2% O_2_, right) by MTT assay. Data are expressed as a percentage of untreated controls and presented as mean ± SEM of no less than three independent experiments. * *p* < 0.05, ** *p* < 0.01 by paired Student’s *t*-test (b); Figure S4: Chemical structures of anthracyclines used in this study (adapted from Menna et al., 2007 [12]). Representative structures of doxorubicin (DOX) (a) and aclarubicin (ACLA) (b) are shown. DOX contains a monosaccharide moiety (R) whereas ACLA carries a trisaccharide substituent (R1). Notably, in contrast to DOX, ACLA lacks the hydroquinone moiety within the tetracyclic anthracycline ring system (indicated by **), a structural feature critical for redox cycling and superoxide generation; Figure S5: Hypoxia reveals myoglobin-dependent differences in doxorubicin sensitivity and intracellular doxorubicin fluorescence. MDA-MB-468 myoglobin knockout (MBKO) cells transfected with (*mCherry*/*mCherryMB*) vectors cells were treated with doxorubicin (DOX, 40 nM) for 72 h under normoxic (21% O_2_) or hypoxic (0.2% O_2_) conditions. Cell viability was assessed by MTT assay. Data are expressed as a percentage of untreated controls (a). Intracellular DOX levels were quantified by flow cytometry and expressed as the geometric mean fluorescence intensity (X-Gmean) (b). Each data point represents an independent experiment; different symbol shapes are used only to distinguish the experimental groups shown on the x-axis. The horizontal line indicates the group mean. * *p* < 0.05, ** *p* < 0.01 by paired Student’s *t*-test; Figure S6: Myoglobin is associated with increase cellular complexity in hypoxic breast cancer cells. MDA-MB-468 wild-type (WT) and myoglobin-knockout (MBKO) cells (a); MDA-MB-468 cells transfected with *mCherry* or *mCherryMB* vectors (b), were incubated for 72 h under normoxic (21% O_2_) or hypoxic (0.2% O_2_) conditions. Cellular granularity/complexity was assessed by side scatter height (SSC-H), expressed as geometric mean (X-GMean). Data are presented as mean ± SEM (n = 6), with each dot representing an independent experiment. Horizontal lines indicate group means. Each data point represents an independent experiment; different symbol shapes are used only to distinguish the experimental groups shown on the x-axis. The horizontal line indicates the group mean. Statistical significance was determined using a paired Student’s *t*-test (* *p* < 0.05, ** *p* < 0.01, *** *p* < 0.001); Figure S7: High *myoglobin* expression is associated with reduced pathological complete response in dichotomous subgroup analyses across breast cancer cohorts. Exploratory dichotomous analyses were performed using median-based stratification of *MB* expression within each cohort. Forest plot showing cohort-specific and pooled odds ratios (ORs) for pathological complete response (pCR) in *MB*-high versus *MB*-low tumors across GSE25055, GSE25065, GSE20194, and GSE41998 cohorts. Odds ratios and 95% confidence intervals (95% CIs) were derived from logistic regression analyses. Fixed-effect meta-analysis was performed using inverse-variance weighting. OR < 1 indicates lower odds of achieving pCR in *MB*-high tumors. Figure S8: Original Western blot images of Figure S2. The membrane was cut after protein transfer to allow separate immunodetection of β-actin (upper part of the membrane) and myoglobin (low-er part of the membrane).

## Abbreviations

AC	Fluorouracil, doxorubicin, and cyclophosphamide
ACLA	Aclarubicin
ADM	Adrenomedullin
ATCC	American Type Culture Collection
BACTIN	Beta Actin
BC	Breast Cancer
CA9	Carbonic Anhydrase 9
cDNA	Complementary DNA
CI	Confidence Interval
CO	Carbon Monoxide
CO_2_	Carbon Dioxide
CORM-3	Carbon Monoxide-Releasing Molecule-3
CQ	Chloroquine
CTRL	Control
DAPI	4′,6-Diamidino-2-Phenylindole
DMEM DMSO	Dulbecco’s Modified Eagle Medium Dimethyl Sulfoxide
DOX	Doxorubicin
EPR	Electron Paramagnetic Resonance
ER	Estrogen receptor
FAC	Fluorouracil, doxorubicin, and cyclophosphamide
FACS	Fluorescence-Activated Cell Sorting
FBS	Fetal Bovine Serum
FEC	Fluorouracil, epirubicin, and cyclophosphamide
FITC	Fluorescein Isothiocyanate
GEO	Gene Expression Omnibus
GFP	Green Fluorescent Protein
HER2	Human epidermal growth factor receptor 2
IXA	Ixabepilone
LDHA	Lactate Dehydrogenase A
LAMP1	Lysosomal Associated Membrane Protein 1
MB	Myoglobin
MBCO	Carboxymyoglobin
MBIV	Ferrylmyoglobin
MBKO	Myoglobin Knockout
MCHERRY	PmCherry-C1 vector
MCHERRYMB	MCherry-tagged myoglobin
MTT	3-(4,5-Dimethylthiazol-2-yl)-2,5-Dipheny ltetrazolium Bromide
NA	Not available
NDRG1	N-Myc Downstream Regulated Gene 1
OR	Odds Ratio
O_2_	Oxygen
O_2_•^−^	Superoxide Anion
PAM50	Basal-like intrinsic molecular subtype defined by PAM50 gene expression profiling
PBS	Phosphate-Buffered Saline
pCR	Pathological Complete Response
PE	Phycoerythrin
PGAM1	Phosphoglycerate Mutase 1
PR	Progesterone receptor
PTX	Paclitaxel
qRT-PCR	Quantitative Reverse Transcription Polymerase Chain Reaction
RD	Residual Disease
RNA	Ribonucleic Acid
ROS	Reactive Oxygen Species
RPMI	Roswell Park Memorial Institute Medium
RIPA	Radioimmunoprecipitation Assay Buffer
SEM	Standard Error of the Mean
SLC2A1	Solute Carrier Family 2 Member 1
SRB	Sulforhodamine B
SSC-H	Side Scatter Height
TALEN	Transcription Activator-Like Effector Nuclease
*t*-Boc-Ala	*tert*-Butoxycarbonyl-Alanine
TBS	Tris-Buffered Saline
TBST	Tris-Buffered Saline with Tween/Triton
TNBC	Triple- Breast Negative Cancer
VEGFA	Vascular Endothelial Growth Factor A
WT	Wild Type
X-GMEAN	X-Axis Geometric Mean Fluorescence Intensity
