# Supplementary material for: Role of Endogenous Myoglobin in Anthracycline Response in Breast Cancer
Source: Biomolecules. 2026 Jul 18;16(7):1055. doi: 10.3390/biom16071055 (PMC13406783; doi:10.3390/biom16071055)
Supplement: Supplementary file 1 [file biomolecules-16-01055-s001.zip › biomolecules-4418772-supplementary.pdf]

Supplementary Methods

RNA extraction and qRT-PCR

Total RNA was isolated from BC cells using TRI reagent® (Sigma–Aldrich, St. Louis, MO, USA; Cat. No. T9424) and reverse-transcribed into cDNA using the ProtoScript® M-MuLV First Strand cDNA Synthesis Kit (New England Biolabs, Ipswich, MA, USA; Cat. No. E6300). The resulting cDNA was used as a template for quantitative real-time PCR (qRT-PCR). Changes in mRNA expression levels were analyzed in triplicates using Taq-Man™ Gene Expression Assays (Applied Biosystems, Thermo Fisher Scientific, Foster City, CA, USA) specific for *MB* (Hs00193520_m1), *ABCG2* (Hs01053790_m1), and *ABCC2* (Hs00166123_m1). *18S* rRNA (Hs03928985_g1) served as the endogenous normalization control. Relative mRNA expression levels were calculated using the 2^–DCt method.

Western blot

Cell lysates were prepared in ice-cold RIPA buffer [50mM Tris HCl pH 8, 150 mM NaCl, 1mM EDTA, 1% Triton X-100, 0.5% sodium deoxycholate, 0.1% SDS]. Protein concentrations were determined using the Coomassie Plus™ Protein Assay Reagent (Thermo Fisher Scientific, Waltham, MA, USA; Cat. No. 1856210). A total of 95 μg protein from cell lysates were subjected to SDS-PAGE NuPAGE 4-12% Bis-Tris gel (Invitrogen, Thermo Fisher Scientific, Waltham, MA, USA; Cat. No. NP0335BOX) and transferred to polyvinylidene difluoride membranes (Immobilon®-P PVDF Membrane, Merck Millipore, Burlington, MA, USA; Cat. No. IPVH00010). Membranes were blocked with 5% non-fat dry milk (GeneSpin, Milan, Italy; Cat. No. STS-M500) prepared in Tris-buffered saline (TBS) containing 0.1% Triton X-100 (TBST) and then incubated overnight at 4°C with primary antibodies diluted in 3% milk in TBST. Membranes were washed three times with TBST and incubated with the appropriate secondary antibodies for 1 h at room temperature. Following two washes with TBST and one wash with TBS, proteins were detected by enhanced chemiluminescence using Pierce™ ECL Western Blotting Substrate (Thermo Fisher Scientific, Waltham, MA, USA; Cat. No. 32209). The primary antibodies used were a rabbit monoclonal anti-MB antibody (1:1000; clone EP3081Y; Abcam, Cambridge, UK; Cat. No. ab77232) and a horseradish peroxidase-conjugated mouse monoclonal anti-β-actin antibody, used as a loading control (1:30,000; clone AC-15; Sigma-Aldrich, St. Louis, MO, USA; Cat. No. A3854). After two washes with TBST and one wash with TBS, proteins were detected by enhanced chemiluminescence using Pierce™ ECL Western Blotting Substrate (Thermo Fisher Scientific, Waltham, MA, USA; Cat. No. 32209).

Supplementary Figures

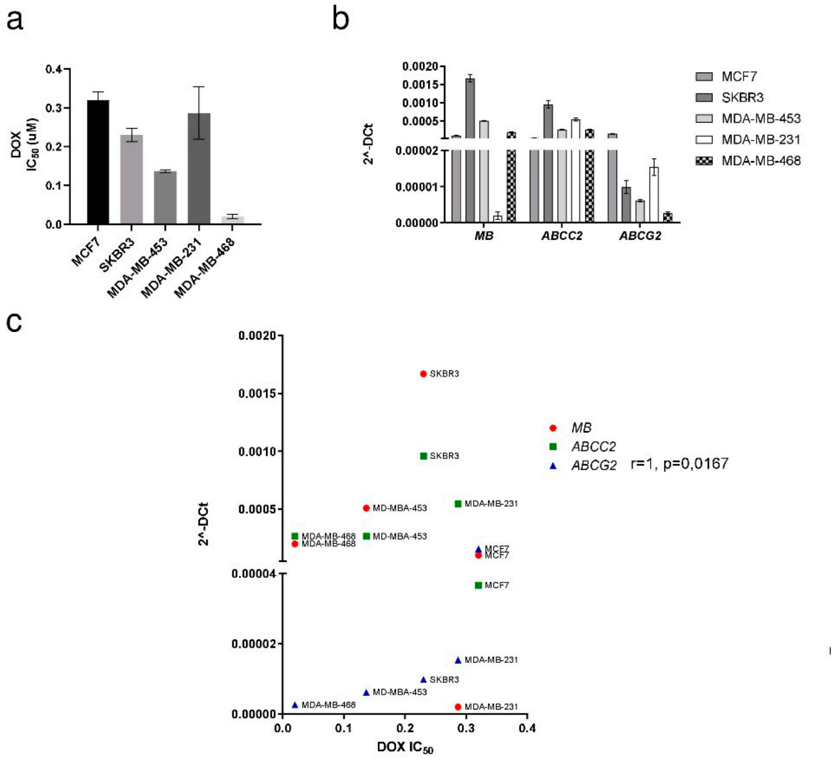

Figure S1. DOX IC_{50} and basal expression of myoglobin in breast cancer cell lines. IC_{50} values of DOX (μM) evaluated in breast cancer cell lines (MCF7, SKBR3, MDA-MB-453,-468,-231) after 72 h of incubation with different doses of DOX; toxicity was measured by sulforhodamine B assay. Each dot in the scatter plot represents an independent experiment. Lines show the mean IC_{50} doses derived from three independent experiments. All the experiments were performed in normoxia (21% O_2) (a). mRNA expression of myoglobin (*MB*), ATP Binding Cassette Subfamily C Member 2 (*ABCC2*) and ATP Binding Cassette Subfamily G Member 2 (*ABCG2*) in breast cancer cell lines (MCF7, SKBR3, MDA-MB-453,-468,-231) was evaluated by qPCR. Results are presented as the mean ± SEM of three independent experiments. 18S was used as housekeeping gene. All experiments were performed in normoxia (21% O2) (b). Correlation analysis between doxorubicin (DOX) IC_{50} values (μM, x-axis) and mRNA expression levels of *MB*, *ABCC2*, and *ABCG2* (y-axis, expressed as 2^-DCt) across a panel of breast cancer cell lines (MCF7, SKBR3, MDA-MB-453, MDA-MB-468, and MDA-MB-231). Associations were assessed using Spearman's rank correlation coefficient (r) (c).

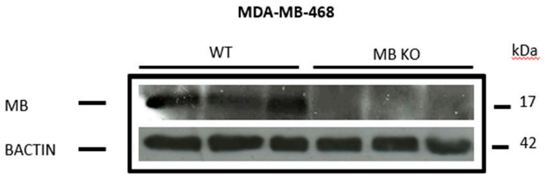

Figure S2. Myoglobin expression in MDA-MB-468 cell line. Western blot analysis of myoglobin (MB) in wild-type (WT) and MB knockout (MBKO) MDA-MB-468 cell lines. Data are representative of three independent cell passages. Beta actin (BACTIN) was used as a loading control.

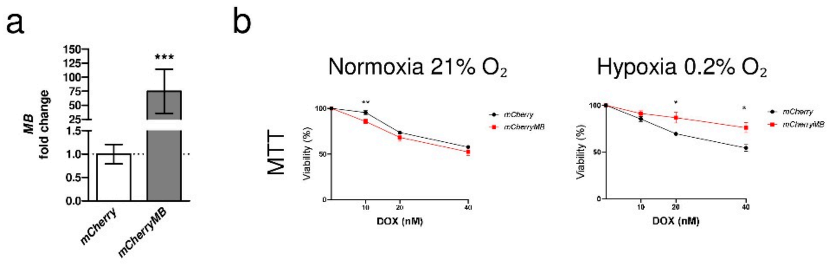

Figure S3. Myoglobin re-expression in MBKO cells confers hypoxia-dependent protection against doxorubicin-induced cytotoxicity. Expression of myoglobin (MB) evaluated by qPCR analysis of myoglobin (MB) mRNA in MDA-MB-468 cell line transfected with empty (*mCherry*) and MB containing (mCherryMB) vectors. 18S was used as housekeeping gene. *** *p <0.001,* by paired Student's *t*-test (a). Effects of various concentrations of doxorubicin (DOX) (10, 20 and 40 nM) on MDA-MB-468 MBKO cells (*mCherry*/*mCherryMB*) viability measured after 72 h of incubation in normoxia (21% O2, left) and hypoxia (0.2% O2, right) by MTT assay. Data are expressed as a percentage of untreated controls and presented as mean ± SEM of no less than three independent experiments. * *p <0.05, **p <0.01* by paired Student's *t*-test (b).

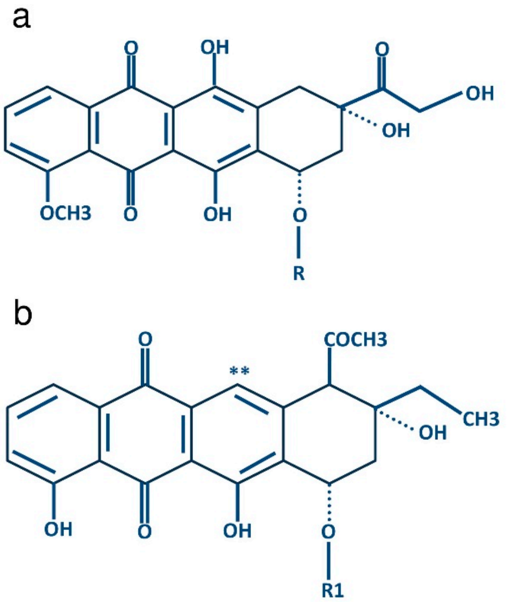

Figure S4. Chemical structures of anthracyclines used in this study (adapted from Menna et al., 2007). Representative structures of doxorubicin (DOX) (a) and aclarubicin (ACLA) (b) are shown. DOX contains a monosaccharide moiety (R) whereas ACLA carries a trisaccharide substituent (R1). Notably, in contrast to DOX, ACLA lacks the hydroquinone moiety within the tetracyclic anthracycline ring system (indicated by **), a structural feature critical for redox cycling and superoxide generation.

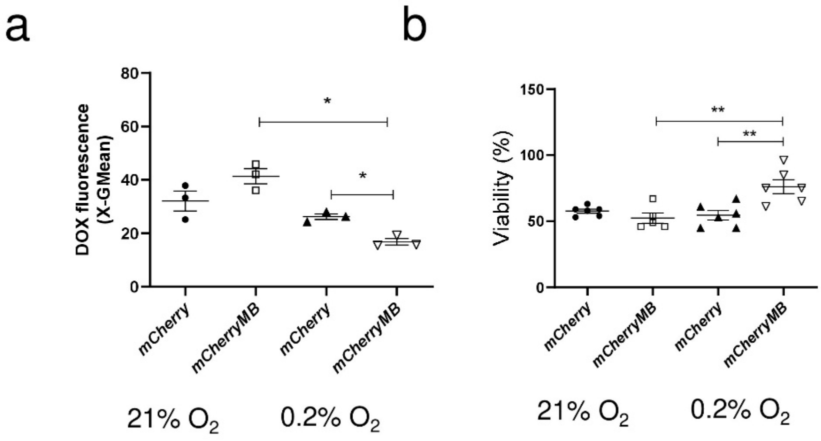

Figure S5. Hypoxia reveals myoglobin-dependent differences in doxorubicin sensitivity and intracellular doxorubicin fluorescence. MDA-MB-468 myoglobin knockout (MBKO) cells transfected with (*mCherry*/*mCherryMB*) vectors cells were treated with doxorubicin (DOX, 40 nM) for 72 h under normoxic (21% O_2) or hypoxic (0.2% O_2) conditions. Cell viability was assessed by MTT assay. Data are expressed as a percentage of untreated controls (a). Intracellular DOX levels were quantified by flow cytometry and expressed as the geometric mean fluorescence intensity (X-Gmean) (b). Each data point represents an independent experiment; different symbol shapes are used to distinguish the experimental groups shown on the x-axis. The horizontal line indicates the group mean. * p *<0.05*, **p *<0.01* by paired Student's t-test.

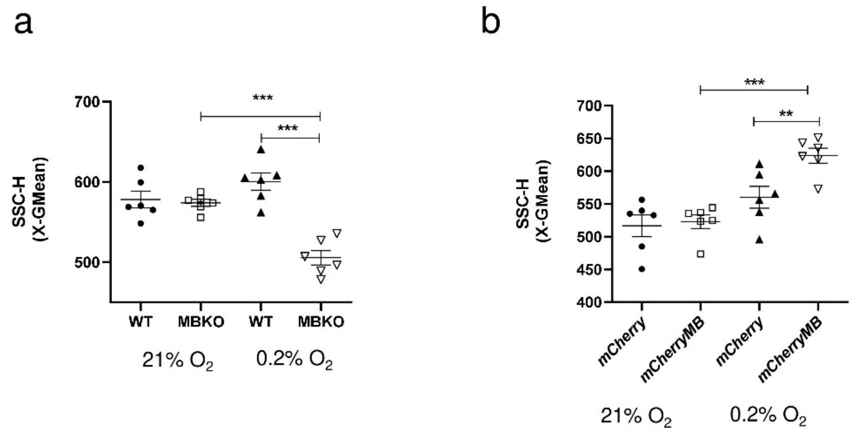

Figure S6. Myoglobin is associated with increase cellular complexity in hypoxic breast cancer cells. MDA-MB-468 wild-type (WT) and myoglobin-knockout (MBKO) cells (a); MDA-MB-468 cells transfected with *mCherry* or *mCherryMB* vectors (b), were incubated for 72 h under normoxic (21% O_2) or hypoxic (0.2% O_2) conditions. Cellular granularity/complexity was assessed by side scatter height (SSC-H), expressed as geometric mean (X-GMean). Data are presented as mean ± SEM (n = 6), with each dot representing an independent experiment. Horizontal lines indicate group means. Each data point represents an independent experiment; different symbol shapes are used to distinguish the experimental groups shown on the x-axis. The horizontal line indicates the group mean. Statistical significance was determined using a paired Student's t-test (*$p < 0.05$, **$p < 0.01$, ***$p < 0.001$).

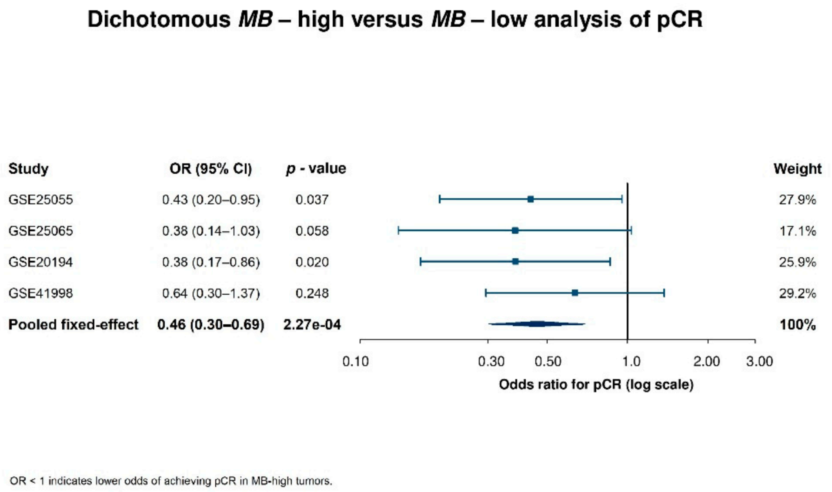

Figure S7. High *myoglobin* expression is associated with reduced pathological complete response in dichotomous subgroup analyses across breast cancer cohorts. Exploratory dichotomous analyses were performed using median-based stratification of *MB* expression within each cohort. Forest plot showing cohort-specific and pooled odds ratios (ORs) for pathological complete response (pCR) in *MB*-high versus *MB*-low tumors across GSE25055, GSE25065, GSE20194, and GSE41998 cohorts. Odds ratios and 95% confidence intervals (95% CIs) were derived from logistic regression analyses. Fixed-effect meta-analysis was performed using inverse-variance weighting. OR < 1 indicates lower odds of achieving pCR in *MB*-high tumors.

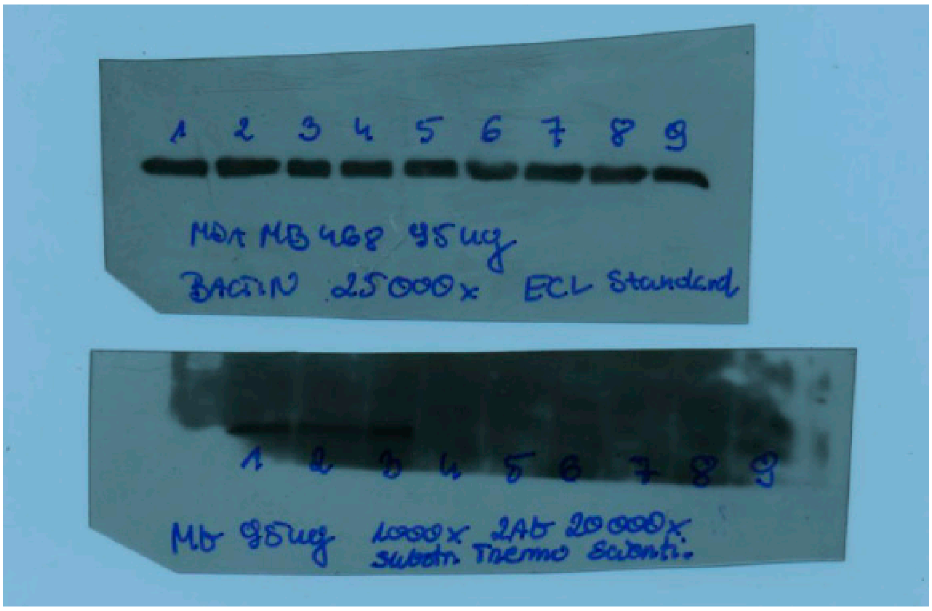

Figure S8. Original Western blot images of *Figure S2*. The membrane was cut after protein transfer to allow separate immunodetection of β-actin (upper part of the membrane) and myoglobin (lower part of the membrane).
